# Understanding sex differences in cardiovascular medicine: a plea for combining clinical trials with real-world data

**DOI:** 10.1007/s12471-024-01894-4

**Published:** 2024-08-08

**Authors:** Eric Boersma

**Affiliations:** Cardiovascular Institute, Thorax Centre, Department of Cardiology, Erasmus Medical Centre, Rotterdam, The Netherlands

As has been demonstrated by multiple studies, relative to the disease prevalence in the general population, women are underrepresented in cardiovascular clinical trials. As regards drug trials, the participation to (population) prevalence ratio (PPR) is well below the generally accepted desirable threshold of 0.8 in the large areas of heart failure, coronary artery disease and acute coronary syndrome [1]. Clinical trials on hypertension and atrial fibrillation (AF) show PPRs for women above 0.8 (and below 1.2), indicating adequate representation, at least in terms of numbers. The classification into disease areas does not dismiss significant outliers either above or below. For example, in the LoDoCo2 colchicine trial in patients with chronic coronary disease, only 15% of the participants were women [2], highlighting substantial underrepresentation. In contrast, female participation reached 60% in the I‑PRESERVE irbesartan trial in heart failure patients with preserved ejection fraction, indicating more balanced representation [3].

In this issue of *The Netherlands Heart Journal*, Ekrami and colleagues report on the participation of female patients at the University Medical Centre Groningen (UMCG), the Netherlands, in eight recent prospective AF studies, mainly clinical trials [4]. It appeared that 42% of the AF patients screened for study participation were women. This figure corresponds well to the estimated 40% population prevalence of AF in the Netherlands in 2019, based on the Global Burden of Disease database, and to the fact that 38% of the UMCG patients with an AF diagnosis in the 2018–2022 period were women. After screening, an equal percentage of women and men were finally included in the AF studies. As a result, female representation approximated that of the general AF population and the hospital AF population. The UMCG investigators are to be congratulated on that achievement.

However, the article by Ekrami et al. does not clarify the success factors that have led to the adequate inclusion of women in their AF trials. Are these related to aspects frequently mentioned in connection with the limited participation of women in cardiovascular trials, including sex differences in disease presentation, awareness, hormonal influences, regulatory issues (such as not including pregnant women to prevent adverse effects on the foetus) and socioeconomic factors? What (logistical) measures have been taken at the UMCG that other clinics can learn from? Moreover, does the UMCG achieve similarly good results in other cardiovascular areas? Or do their findings fit the general pattern of AF trials where, numerically, women seem to be adequately represented? [1]. Perhaps the researchers can provide further information on these aspects.

The discussion about the participation of women in clinical trials should not focus solely on PPRs. Indeed, adequate numerical representation of women (and men) in clinical trials is important. More relevant, however, is the question of how representative the included patients are of the target patient population. Are the 57 women and 104 men that were ultimately included an appropriate reflection of the UMCG AF population? The fact that 75% of the screened patients did not meet the inclusion criteria of the trials suggests that this is not the case. In this regard, there is still much work to be done. Nationwide registry-based initiatives such as the DUTCH-AF Registry, and in particular registry-based clinical trials, might help to close the gap between trial and practice.

To some extent, randomised clinical trials (RCTs) serve as the cornerstone of evidence-based medicine by providing internally valid estimates of treatment efficacy. It is therefore reassuring that systematic reviews of RCTs across various medical disciplines have not revealed differential treatment effects between women and men, including cardiovascular diseases [1, 5, 6]. However, it could be argued that a lack of evidence for a difference is not evidence of absence. In this context, it is important to recognise that RCTs are inherently designed to study effects in homogeneous populations. They are typically underpowered to detect heterogeneous treatment effects. In fact, trials need to be four times larger to detect a sex-treatment interaction of comparable magnitude to the main effect [7], highlighting the challenge of studying more subtle interactions. Therefore, even if the female-to-male ratio were 50:50 (statistically optimal for studying heterogeneity), RCTs alone may not adequately address the need for sex-sensitive individualised treatment. Increasing the participation of women in clinical trials is essential, but additional complementary policies are also needed. In this respect as well, systematic, large-scale, unselected registration of routine practices can make a valuable contribution. Indeed, from a methodological perspective, there is work to be done to obtain unbiased efficacy estimates in target populations by integrating data from RCTs and real-world sources. However, real-world data provide deeper insights into absolute risks, treatment effectiveness and drug side effects, thus thereby revealing differences between women and men in clinical practice. While our era rightly emphasises diversity in medicine, it is crucial to leverage technical solutions for collecting and analysing large-scale diversity data. Let us embrace these solutions to advance healthcare for all.
